# Combining chitin biological conduits with small autogenous nerves and platelet‐rich plasma for the repair of sciatic nerve defects in rats

**DOI:** 10.1111/cns.13640

**Published:** 2021-04-10

**Authors:** Chang‐Feng Lu, Bo Wang, Pei‐Xun Zhang, Shuai Han, Wei Pi, Yu‐Hui Kou, Bao‐Guo Jiang

**Affiliations:** ^1^ Department of Orthopedics and Trauma, Key Laboratory of Trauma and Neural Regeneration (Ministry of Education/Peking University Peking University People’s Hospital Beijing 100044 China

**Keywords:** peripheral nerve defect, peripheral nerve repair, platelet‐rich plasma, small autologous nerve

## Abstract

**Aims:**

Peripheral nerve defects are often difficult to recover from, and there is no optimal repair method. Therefore, it is important to explore new methods of repairing peripheral nerve defects. This study explored the efficacy of nerve grafts constructed from chitin biological conduits combined with small autogenous nerves (SANs) and platelet‐rich plasma (PRP) for repairing 10‐mm sciatic nerve defects in rats.

**Methods:**

To prepare 10‐mm sciatic nerve defects, SANs were first harvested and PRP was extracted. The nerve grafts consisted of chitin biological conduits combined with SAN and PRP, and were used to repair rat sciatic nerve defects. These examinations, including measurements of axon growth efficiency, a gait analysis, electrophysiological tests, counts of regenerated myelinated fibers and observations of their morphology, histological evaluation of the gastrocnemius muscle, retrograde tracing with Fluor‐Gold (FG), and motor endplates (MEPs) distribution analysis, were conducted to evaluate the repair status.

**Results:**

Two weeks after nerve transplantation, the rate and number of regenerated axons in the PRP‐SAN group improved compared with those in the PRP, SAN, and Hollow groups. The PRP‐SAN group exhibited better recovery in terms of the sciatic functional index value, composite action potential intensity, myelinated nerve fiber density, myelin sheath thickness, and gastrectomy tissue at 12 weeks after transplantation, compared with the PRP and SAN groups. The results of FG retrograde tracing and MEPs analyses showed that numbers of FG‐positive sensory neurons and motor neurons as well as MEPs distribution density were higher in the PRP‐SAN group than in the PRP or SAN group.

**Conclusions:**

Nerve grafts comprising chitin biological conduits combined with SANs and PRP significantly improved the repair of 10‐mm sciatic nerve defects in rats and may have therapeutic potential for repairing peripheral nerve defects in future applications.

## Graphical abstract

Schematic shows a novel method on treating peripheral nerve defect. The nerve grafts comprising chitin biological conduits combined with SANs and PRP can enhance the repair of 10‐mm sciatic nerve defects in rats. PRP and SANs in the graft are completely self‐derived and obtained quickly and safely.

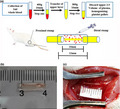

## 1 INTRODUCTION

Peripheral nerve damage often leads to pain and severe disability. In the United States, approximately 50,000 people undergo surgery to repair peripheral nerve damage each year. The causes of injury include traffic accidents, fractures, explosive injuries, and iatrogenic injuries. Only a small proportion of these patients achieve full functional recovery after treatment.^1^, ^2^, ^3^, ^4^, ^5^, ^6^ When the peripheral nerve defect is larger than 3 cm, the current gold standard clinical treatment is autogenous nerve transplantation. The main problems with autogenous nerve transplantation are that it requires the use of two surgical sites and the donor area is left denervated. Many new therapeutic strategies to improve nerve repair are being developed in basic, preclinical, and clinical trials. Tissue‐engineered nerve grafts exhibit the most potential as a replacement for autogenous nerve grafts and are widely used to repair peripheral nerve defects.^7^, ^8^, ^9^, ^10^

The core factors for constructing tissue‐engineered nerve grafts include biological scaffolds, seed cells, and various growth factors.^11^ Most tissue‐engineered nerve grafts are constructed from nerve conduits. Nerve conduits grafted between nerve defects provide a suitable microenvironment for regenerating axons, thus improving the efficiency of nerve regeneration and restoring impaired motor and sensory functions. Chitin biological absorbable conduits exhibit good biological properties in the peripheral nerve defect repair model.^12^, ^13^ A single proximal axon produces multiple lateral buds during nerve regeneration, a phenomenon known as the “multiple amplification” effect, which leads to a maximum power rate of approximately 3.3 for nerve regeneration.^14^ More importantly, small autogenous nerves (SANs) secrete neurotrophic factors and promote Schwann cell (SC) proliferation during Wallerian degeneration.^15^, ^16^ SANs with a complete structure provide a hierarchically aligned structure and an optimal bridge for the rapid growth of axons. Therefore, tissue‐engineered nerve grafts can be constructed using SANs. In addition, the application of SANs overcomes the problem of insufficient sources for autologous nerve transplantation by increasing the supply of autogenous nerves and reduces the level of denervation at the donor nerve site.^17^, ^18^, ^19^

Neurotrophic factors are important components of tissue‐engineered nerve grafts. Soluble neurotrophic factors can be directly integrated into nerve conduits. These nutritional factors include nerve growth factor (NGF), insulin‐like growth factor (IGF), fibroblast growth factor, brain‐derived neurotrophic factor (BDNF), and glial cell line‐derived neurotrophic factor (GDNF).^20^, ^21^, ^22^ Platelet‐rich plasma (PRP) contains a variety of neurotrophic factors and cytokines that participate in the regulation of early inflammation, angiogenesis, fibrogenesis, and macrophage polarization during repair. Platelet‐derived growth factors, such as transforming growth factor‐microRNA, IGF‐1, platelet‐derived growth factor‐AB, vascular endothelial growth factor, GDNF, NGF, and platelet‐derived body signal molecules such as microRNA, promote the distal growth of axons and accelerate the migration and proliferation of SCs.^23^, ^24^ All of these processes are key to nerve functional recovery. Moreover, activated PRP is a gel and can be used as a framework for nerve regeneration. Thus, PRP provides the appropriate biomimetic microenvironment for nerve regeneration.^25^, ^26^

Because of the potential roles PRP and SANs play in promoting nerve regeneration, nerve grafts were constructed from chitin biological conduits combined with SANs and PRP to repair 10‐mm sciatic nerve defects in rats in this study. The effectiveness of nerve injury repair was systematically evaluated using animal experimental methods.

## 2 MATERIALS AND METHODS

### 2.1 Preparation of chitosan neural conduits

Each chitin biological conduit was prepared according to a previously described protocol (National Invention patent no. ZL01136314.2: Partial deacetylated chitin biological conduit guiding and promoting effective nerve regeneration and its manufacturing method).^12^, ^18^

### 2.2 Preparation and injection of autogenous PRP

**FIGURE 1:**
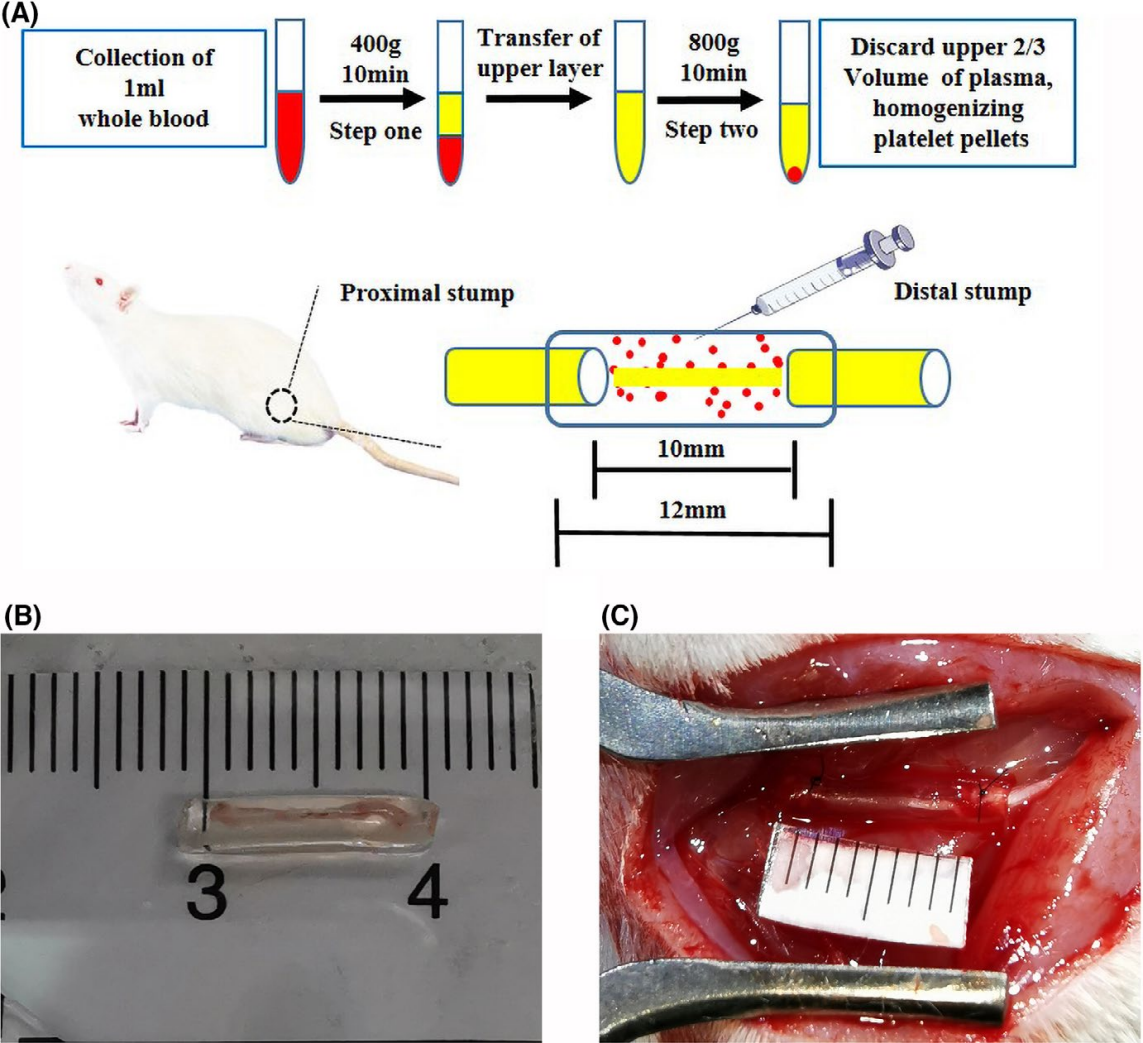
Representation of the materials and methods used in this study. (A) Flowchart representing the preparation of platelet‐rich plasma (PRP) and the experimental diagram representing the surgical process. (B) Representative photograph of a chitin biological conduit combined with a small autogenous nerve. (C) Representative photograph of nerve graft bridging

After 1 week of acclimation, the rats were anesthetized with 2.5% isoflurane gas, and 1 ml of whole blood was collected from the posterior orbital venous plexus of each rat and placed in a collection vessel containing 3.8% (w/v) sodium citrate. The whole blood was centrifuged at 400 × g and 22℃ for 10 min and divided into three layers thereafter. The bottom layer comprised red blood cells, the upper layer comprised acellular plasma, and the middle layer comprised platelets and was known as the white membrane. The platelet‐containing plasma was transferred to a sterile centrifuge tube without anticoagulant and centrifuged at 800× g for 10 min to obtain the platelet concentrate. The upper two‐thirds of the plasma were removed, leaving the lower one‐third that comprised approximately 50 µl of PRP, which was resuspended by gently shaking in a centrifuge tube (Figure 1A). The red blood cells, white blood cells, and platelets in the whole blood and PRP were counted using an automatic counter. The PRP was activated with 10% calcium chloride (Sigma‐Aldrich, St. Louis, MO, USA) and a bovine thrombin (Sigma‐Aldrich) mixture to obtain the PRP gel.

### 2.3 Experimental animals and groups

All animal care and experimental procedures were conducted following the Guidelines for Ethical Review of Experimental Animals for Animal Welfare in China and approved by the Ethics Committee of Peking University People's Hospital, Beijing, China (approval no. 2011–2016). The animal data were reported following the ARRIVE 2.0 guidelines.^27^

Sixty healthy adult female Sprague–Dawley rats (weight, 200–220 g) were purchased from Beijing Vitonlihua Experimental Animal Technology Co., Ltd. (license no: SCXK (Jing) 2016–0006; Beijing, China) and randomly divided into five groups using random number table according to the reported methods: One group received hollow chitin biological conduits (Hollow group), one received chitin biological conduits with SANs (SAN group) (Figure 1B), one received chitin biological conduits with PRP (PRP group), one received chitin biological conduits with PRP and SANs (PRP‐SAN group), and the last group received autografts (Autograft group). All experimental animals were anesthetized with 2.5% isoflurane, and the hair on the right lower extremity was shaved to expose the right sciatic nerve. A 10‐mm defect was created in the right sciatic nerve at the same site. For each rat, the defect was bridged with a chitin biological conduit in the Hollow treatment, a chitin biological conduit containing a small branch of the sciatic nerve in the SAN treatment, a chitin biological conduit injected intravenously with prepared PRP gel in the PRP treatment, a chitin biological conduit containing a small branch of the sciatic nerve and injected with prepared PRP gel in the PRP‐SAN treatment (Figure 1C), or by snipping the sciatic nerve and suturing the epineurium *in situ* in the Autograft treatment. All rats recovered after the operation and were examined at a predetermined time point. The number of SD rats for all the tests per group was shown in Table 1.

**TABLE 1:** Number of SD rats per group

Evaluation item	Hollow	SAN	PRP	PRP‐SAN	Autograft
Histochemical staining of regenerated nerve fibers	3	3	3	3	3
Gait analysis^a^	5	5	5	5	5
Electrophysiological detection^a^	5	5	5	5	5
Morphological evaluation of regenerated nerve fibers^a^	5	5	5	5	5
Immunofluorescence staining of regenerated nerve fibers^b^	2	2	2	2	2
Gastrocnemius recovery^a^	5	5	5	5	5
Fluor‐Gold (FG) retrograde tracing	4	4	4	4	4
The number of MEPs^b^	2	2	2	2	2
Total number	12	12	12	12	12

^a^ The same 5 rats were used in these tests.

^b^ The 2 samples were randomly chosen from the 5 rats mentioned above.

#### 2.3.1 Histochemical staining of regenerated nerve fibers

Three rats in each group were randomly euthanized with excess sodium pentobarbital solution at 3 weeks after nerve transplantation, and the nerve grafts were extracted. After fixation, the nerve grafts were cut into 12‐µm longitudinal sections using a frozen‐section machine. Six slides from each group underwent immunofluorescence staining (NF200/S100/DAPI) and hematoxylin and eosin (HE) staining. A vertical scanner (Zeiss, Jena, Germany) was used to observe the regeneration of the nerve fibers and inflammation at 200× magnification. The sections were stained with NF200 (Sigma‐Aldrich) to reveal the regenerated axons and with S100 (Sigma‐Aldrich) to show SCs, following a previously described method. Briefly, the specimens were incubated with NF200 and S100 primary antibodies overnight at 4℃ and washed three times with PBS for 5 min. The mixed secondary antibodies Alexa488 and Alexa594 (Abcam, Cambridge, UK) were added, and the specimens were incubated at room temperature for 1 h and then dyed with DAPI for 5 min.

#### 2.3.2 Gait analysis

The CatWalk XT 10.6 gait analysis system (Noldus, Wageningen, the Netherlands) was used to evaluate the recovery of muscle motor function at 2, 4, 6, 8, 10, and 12 weeks after nerve transplantation. The animals in each group were placed in a closed passage consisting of a glass platform and black plastic walls. The sciatic functional index (SFI) was calculated using the following formula:

$$\text{SFI}\;=\frac{109.5\;(\text{ETS}‐\text{NTS)}}{\text{NTS}}‐\frac{38.3\;(\text{EPL}‐\text{NPL)}}{\text{NPL}}+\frac{13.3\;(\text{EITS}‐\text{NITS})}{\text{NITS}}‐8.8$$

where ETS is the experimental distance from the first to the fifth toe, NTS is the normal first‐to‐fifth‐toe distance, EPL is the experimental distance from the heel to the top of the third toe, NPL is the normal heel‐to‐third‐toe distance, EITS is the experimental distance from the second to the fourth toe, and NITS is the normal second‐to‐fourth‐toe distance.

#### 2.3.3 Nerve electrophysiological detection

The rats in each group were tested with a neurophysiological instrument (Keypoint, Nørresundby, Denmark) under general anesthesia at 12 weeks after nerve transplantation to determine the recovery of sciatic nerve conduction at the affected side. The gastrocnemius and sciatic nerve at the affected side were carefully exposed during the operation. Two stimulating electrodes were placed at the proximal and distal ends of the nerve graft, and a recording electrode was inserted into the abdomen of the gastrocnemius. Compound muscle action potentials (CMAPs) when the nerve was electrically stimulated at 3.0 mA and 1 Hz were recorded. The delay and peak amplitude of the CMAPs were compared among the different groups.

#### 2.3.4 Morphological evaluation of regenerated nerve fibers

The experimental animals were sacrificed by intraperitoneal injection of an excess of 3% pentobarbital sodium solution at 12 weeks after nerve transplantation. The 5‐mm middle segment of the regenerated nerve and the portion located 2 mm away from the distal end of the nerve graft were obtained. The middle segment of the nerve graft was crosscut for immunofluorescence staining (NF200/S100/DAPI), using the procedure described in section 2.3.1. The distal 2 mm of the regenerated nerve was fixed in 2.5% (v/v) glutaraldehyde for 6 hours after the adherent tissue was trimmed and then fixed with 1% osmium tetroxide. The specimens were cut into 1‐µm‐thick semi‐thin cross‐sectional slices and 70‐nm‐thick ultrathin cross‐sectional slices with a Leica EM UC7 ultramicrotome (Wetzlar, Germany). The specimens were stained with 1% (w/v) toluidine blue/1% (w/v) borax solution for 5 min, and then with lead citrate and uranyl acetate. The images were collected using a BX51 microscope with a DP71 camera (Olympus, Tokyo, Japan). Five sections were randomly selected in each group, and five fields were randomly selected from each section to count the mean density of myelinated nerve fibers by IPP 6.0 observed at 400× magnification. The ultrathin sections were observed using transmission electron microscopy (Philips, Best, The Netherlands). Five sections were randomly selected in each group, and five fields were randomly selected from each section to measure the mean diameter of myelinated nerve fibers and the mean thickness of the myelin sheath by IPP 6.0 at 5000× magnification.

#### 2.3.5 Gastrocnemius recovery

The gastrocnemius of the affected and healthy sides was removed and weighed at 12 weeks after nerve transplantation. The affected gastrocnemius muscles from each group were immersed in 4% paraformaldehyde, embedded in paraffin, and cut into 7‐µm slices. All sections were stained with Masson's trichrome stain. Six sections were randomly selected in each group, and five fields were randomly captured at 200× magnification in each section. The cross‐sectional area of the muscle fibers in each sample was quantitatively analyzed using Image‐Pro Plus 6.0 software (Media Cybernetics, Silver Spring, MD, USA). The wet weight ratio of the gastrocnemius muscles on both sides and the mean cross‐sectional area of the muscle fibers on the affected side were calculated.

#### 2.3.6 Fluor‐Gold (FG) retrograde tracing

The rats were anesthetized with 2.5% isoflurane gas at 12 weeks after nerve transplantation, and sciatic nerves were isolated from the original incision. Each nerve was cut at 5 mm beyond the distal nerve graft, and the proximal ends were soaked in 4% FG solution (Thermo Fisher, Waltham, MA, USA) for 2 hours. The rats were fed for 1 week after the incision was closed and then underwent full heart perfusion fixation with 0.9% sodium chloride solution and 4% paraformaldehyde under anesthesia. The L4–L6 spinal cord and dorsal root ganglion were harvested and soaked in 4% paraformaldehyde solution at 4℃ overnight. The fixed specimens were transferred to a gradient sucrose solution for dehydration (from 10% to 20% to 30%), and all specimens were transferred to the next solution level after the specimens sank. After the specimens sank to the bottom of the 30% sucrose solution, they were embedded in OCT compound and cut into sections while continuously frozen. The spinal cord specimens were cut into 25‐µm‐thick sections, and the dorsal root ganglion (DRG) was cut into 20‐µm‐thick sections. The DRG and spinal cord sections were observed at 200× magnification under a laser confocal microscope (Leica) to assess whether there were FG fluorescence signals in the neuron bodies. The number of motor neurons and DRG sensory neurons in the anterior horn of the spinal cord was counted to evaluate the effects of each treatment on nerve regeneration.

#### 2.3.7 Tissue optical clearing

The rats were anesthetized with 2.5% isoflurane gas and injected with alpha bungarotoxin 647 (BTX 647; Invitrogen, Carlsbad, CA, USA) via the tail vein at 12 weeks after nerve transplantation. The entire heart was fixed by perfusion with 0.9% sodium chloride solution and 4% paraformaldehyde under anesthesia. The bilateral flexor digitorum longus was harvested from each rat and soaked in 4% paraformaldehyde solution at 4℃ for 12 hours. The specimens were washed three times with PBS to remove the formaldehyde and processed until they were transparent to light according to the 3DISCO method. The number of MEPs was analyzed using photomicrographs (LaVision, Göttingen, Germany) and Imaris software (Oxford Instruments, Zurich, Switzerland). Fluorescence signals from BTX 647 binding to the acetylcholine receptor were analyzed at an excitation wavelength of 633 nm, and sample images were collected at 2.52× magnification for subsequent processing and analysis. Three‐dimensional reconstructed images were prepared according to the scanned images using Imaris software.

### 2.4 Data analyses

Blinding was used for all image analysis and behavior assessments. SPSS 22 (SPSS Inc., Chicago, IL, USA) and Origin 8.5 software (Origin Lab, Northampton, MA, USA) was used to process the results. The normality test was performed using Shapiro–Wilk test in SPSS. Normally distributed data were expressed as mean ±standard deviation (SD), while the non‐normally distributed data were expressed as median (interquartile range, IQR). Student's t‐test was performed to compare differences between two groups, and one‐way analysis of variance (ANOVA) was used to compare differences between multiple groups. Tukey's post hoc test was applied when *p*‐value >.05 in the test of homogeneity of variances; otherwise, Dunnett’ s T3 post hoc test was applied. A *p*‐value <.05 was considered to denote statistical significance.

## 3 RESULTS

### 3.1 PRP characteristics

Rat whole‐blood and PRP cell counts are summarized in Table 2. Relatively few white blood cells and red blood cells are found in PRP. The mean platelet count in whole blood was 918.74 ± 125.83 × 10^3^/L and that in PRP was 4,495.94 ± 528.46 × 10^3^/L. Platelets were enriched to a degree of 4.89, with the enrichment degree referring to the ratio of platelet concentration in PRP to that in whole blood.

**TABLE 2:** PRP characteristics

Cell type	Platelet (10^3^/μL, means ±SD)	RBC (10^6^/μL, means ±SD)	WBC (10^3^/μL, means ±SD)
Whole blood	918.74 ± 125.83	7.35 ± 1.36	8.78 ± 1.56
PRP	4,495.94 ± 528.46	1.42 ± 0.25	1.91 ± 0.34

Abbreviations: PRP, platelet‐rich plasma; RBC, red blood cells; WBC, white blood cells.

### 3.2 Chemical staining of regenerated nerve tissue

**FIGURE 2:**
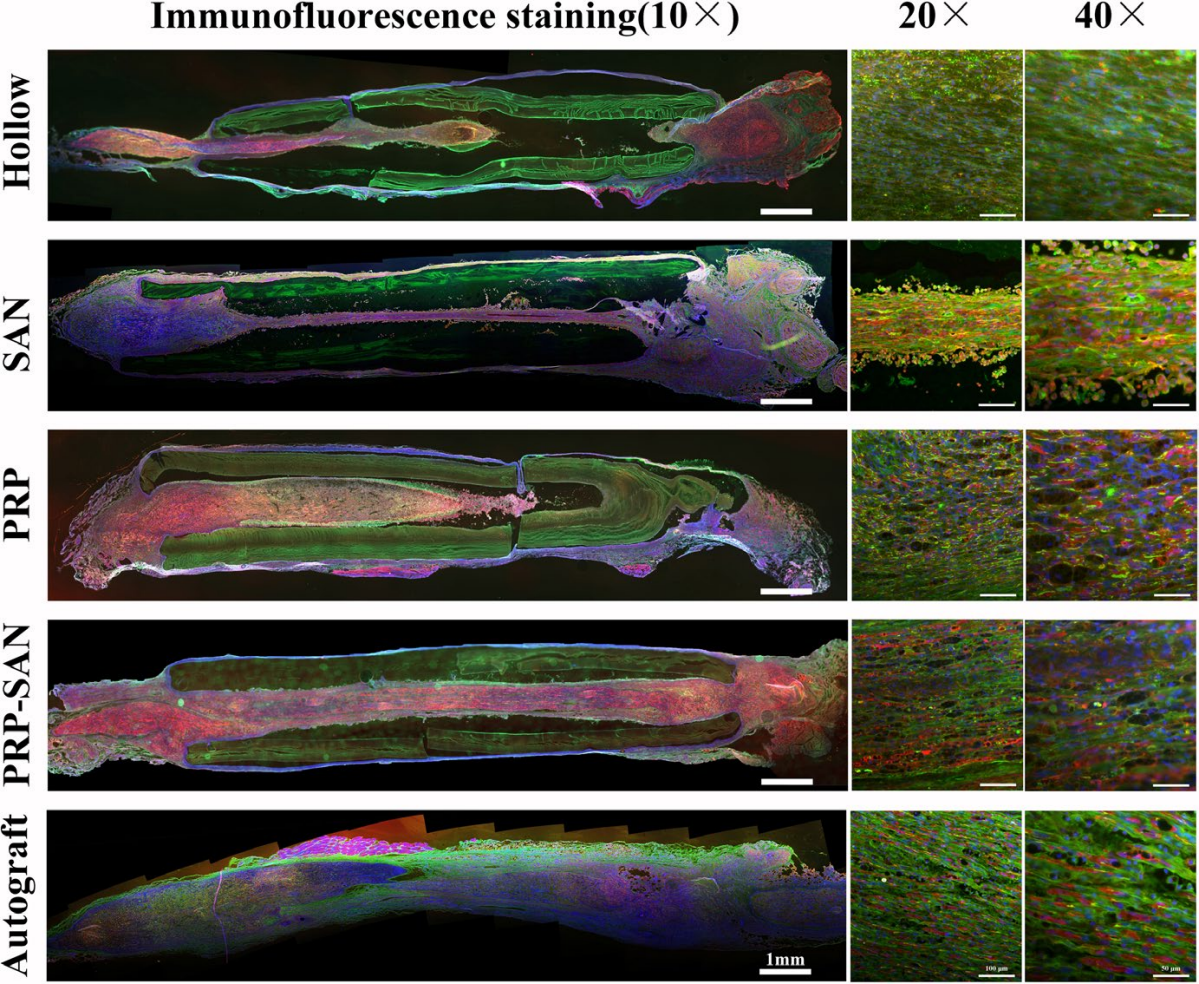
Representative photograph of immunofluorescence staining of nerve grafts from each group at 2 weeks after surgery. Green fluorescence (NF200) indicates axons, red fluorescence (S100) indicates Schwann cells (SCs), and blue fluorescence (DAPI) indicates nuclei

**FIGURE 3:**
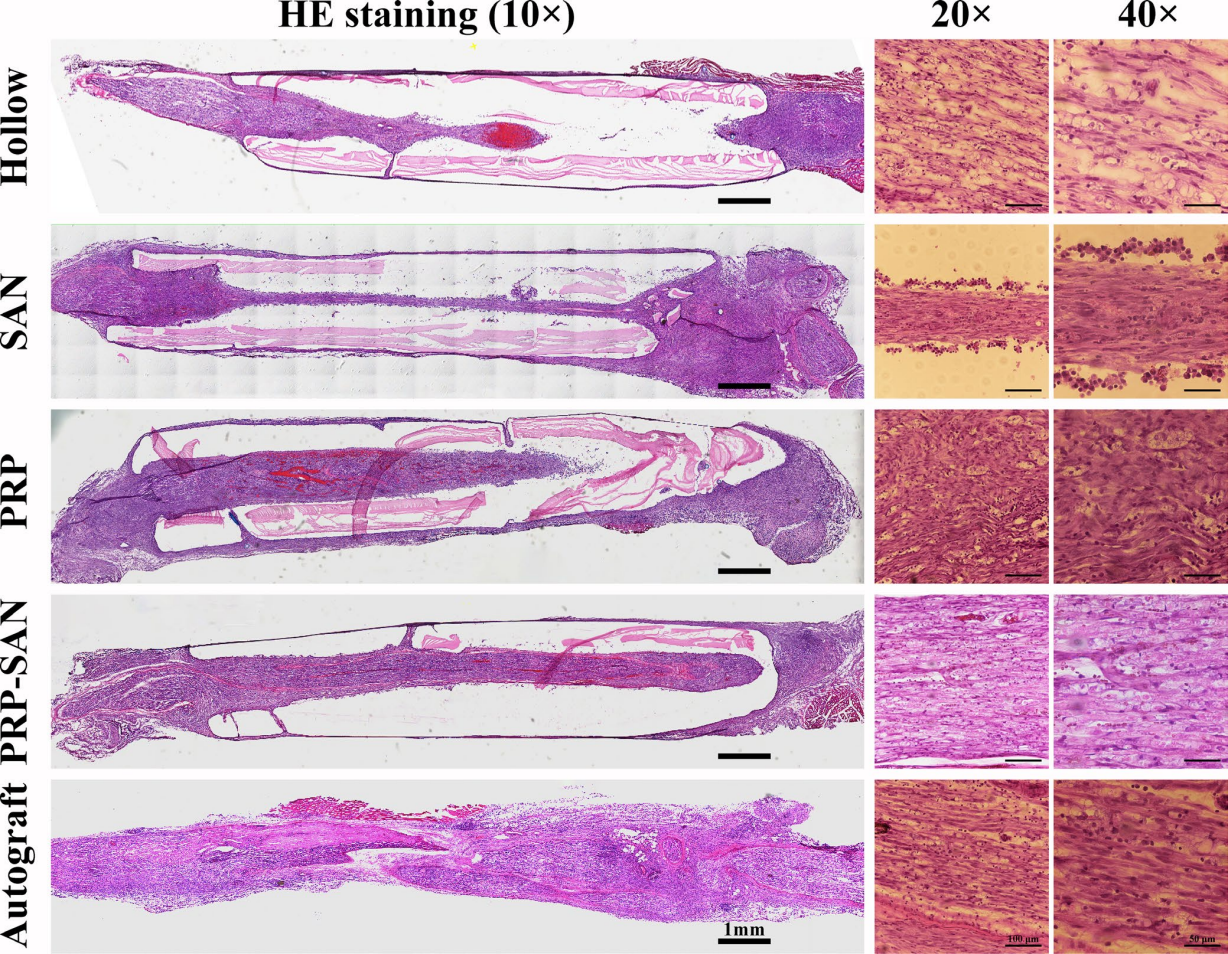
Representative photograph of hematoxylin and eosin staining of the longitudinal sections of nerve grafts from each group at 2 weeks after surgery

Representative results of immunofluorescence (NF200/S100/DAPI) and HE staining of nerve grafts for each group at 3 weeks after nerve transplantation are shown in Figures 2 and 3, respectively. The immunofluorescence staining showed that green fluorescent nerve fibers (NF200 staining) were visible in all groups. Images suggested that the regenerated nerve fibers in the PRP‐SAN and SAN groups were close in length and diameter to those in the Autograft group and obviously longer and thicker, respectively, than those in the PRP and Hollow groups. The images also exhibited that the wall of each chitosan nerve conduit was thinner than before transplantation. The HE staining could draw a similar conclusion with immunofluorescence staining, besides the results revealed no obvious inflammatory cell infiltration in any of the groups.

### 3.3 Gait analysis

**FIGURE 4:**
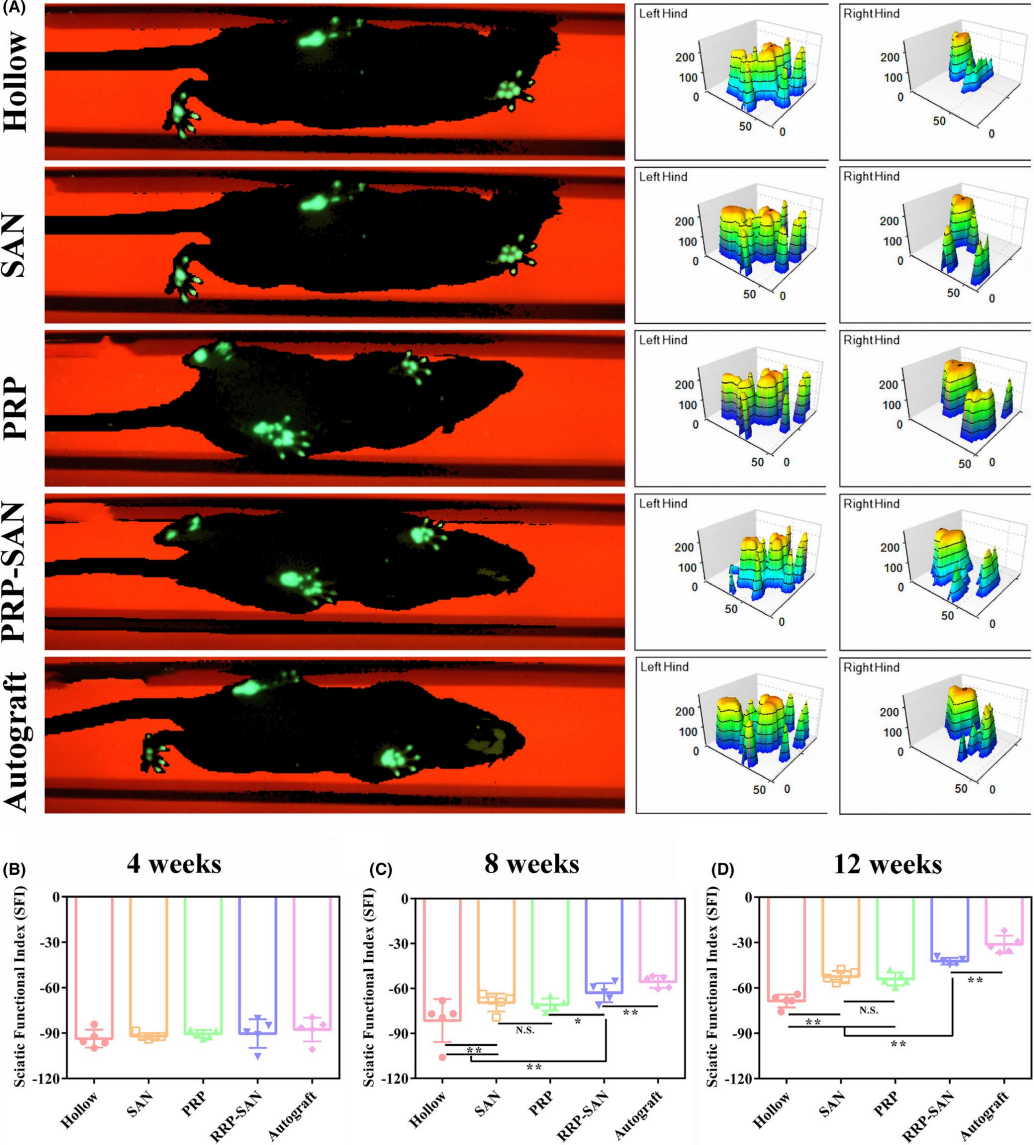
Motor function evaluation at 4, 8, and 12 weeks after surgery. (A) Representative footprints and three‐dimensional stress diagrams for each group at 12 weeks after surgery. The sciatic nerve function index (SFI) in each group at 4 (B), 8 (C), and 12 (D) weeks after surgery. Data are presented as the mean ±standard deviation (SD). ^*^ *p* <.05; ^**^ *p* <.01; N.S., no significant difference

Gait analysis was performed in each group on weeks 4, 8, and 12 after nerve transplantation to evaluate the recovery of motor function, and the results are shown in Figure 4. Figure 4A shows the representative footprints and three‐dimensional footprints of each group at 12 weeks after nerve transplantation. Figure 4B–4D shows the SFI of each group at each time point. The SFI in each group increased gradually with longer postoperative recovery time, and no significant differences were observed in SFI values among groups at 4 weeks after surgery. The SFI of the PRP‐SAN group was higher than those of the PRP, SAN, and Hollow groups at 8 and 12 weeks after surgery (*p *< .05). The SFI values of these four groups were significantly lower than that of the Autograft group (*p *< .01), and no difference was detected between the PRP and SAN groups.

### 3.4 Electrophysiology test

**FIGURE 5:**
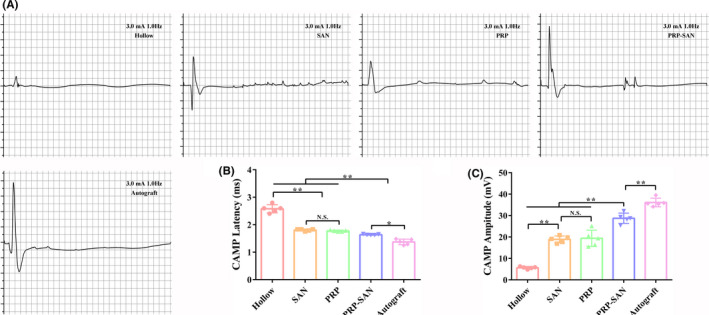
Evaluation of regenerative nerve conduction function at 12 weeks after surgery. (A) Representative photograph of the compound muscle action potentials (CMAPs) in each group. (B) CMAP latency value of each group. (C) CMAP values of each group. Data are presented as the mean ±SD. ^*^ *p* <.05; ^**^ *p* <.01; N.S., no significant difference

The rats in each group were subjected to electrophysiological tests at 12 weeks after nerve transplantation to assess the recovery of nerve conduction, as shown in Figure 5. The CAMP peak amplitude is related to the number of innervated muscle fibers, whereas CAMP delay time is related to the myelin sheath thickness of regenerated nerves. Figure 5A shows the representative CMAP waveforms for each group. Although the CMAP peak amplitude in the PRP‐SAN group was significantly lower than that in the Autograft group (*p *< .01), it was higher than those in the PRP, SAN, and Hollow groups (*p *< .01) (Figure 5C). In addition, the CMAP delay time in the PRP‐SAN group was less than that in the Autograft group (*p *< .05), but significantly greater than those in the PRP, SAN, and Hollow groups (*p *< .01) (Figure 5B).

### 3.5 Degree of axon regeneration and myelination

**FIGURE 6:**
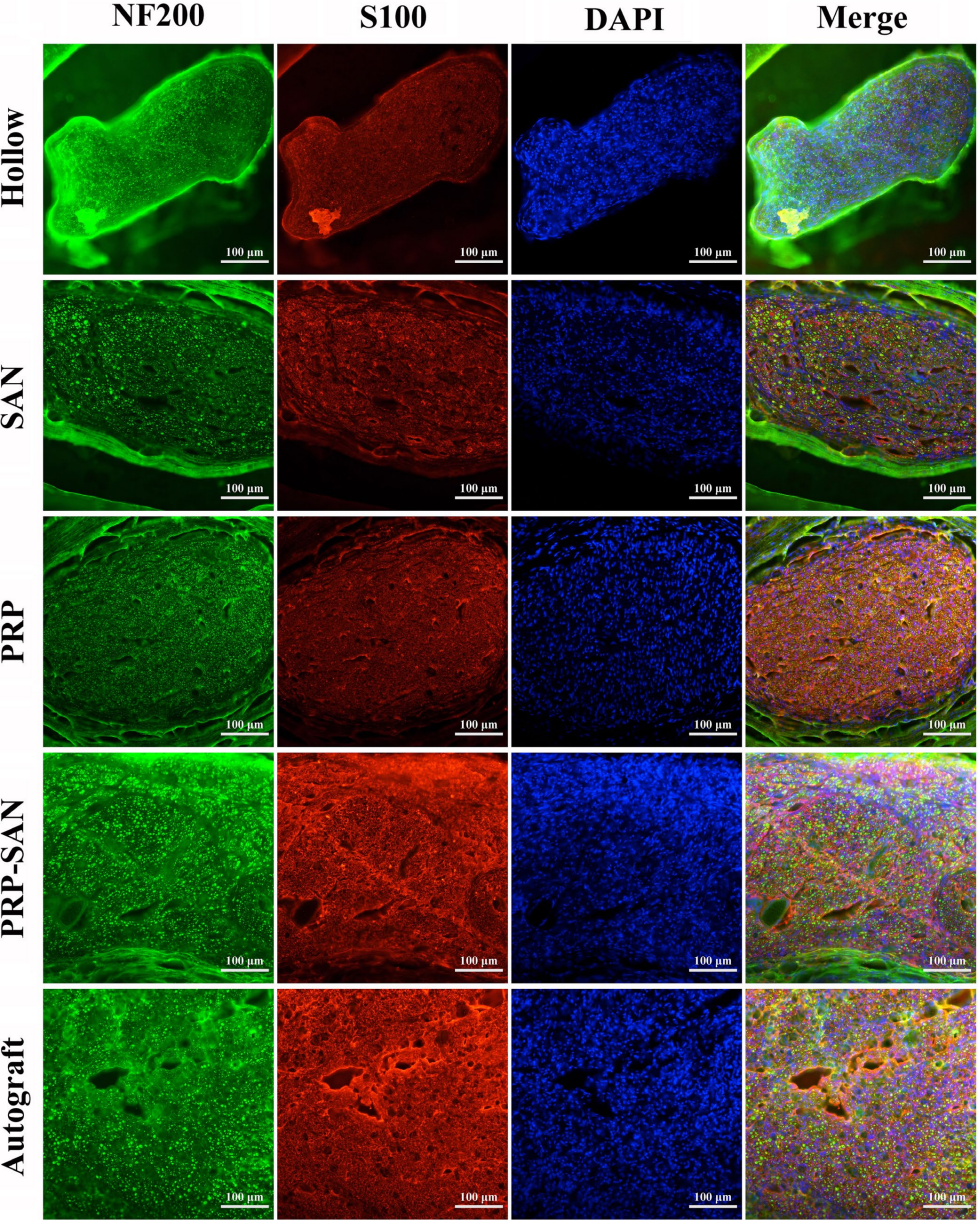
Representative photograph of immunofluorescence staining of the midpoint of a nerve graft from each group at 12 weeks after surgery. Green fluorescence (NF200) shows axons, red fluorescence (S100) shows Schwann cells (SCs), and blue fluorescence (DAPI) shows the nucleus. Scale bar =100 μm

Immunofluorescence staining of the middle cross‐sections of the rat grafts in each group was performed at 12 weeks after nerve transplantation, and the results are shown in Figure 6. Green fluorescence represents regenerating axons, red fluorescence represents SCs, and blue fluorescence represents nuclei. Our observations revealed that more regenerated axons were found in the Autograft and PRP‐SAN groups than in the PRP, SAN, and Hollow groups.

**FIGURE 7:**
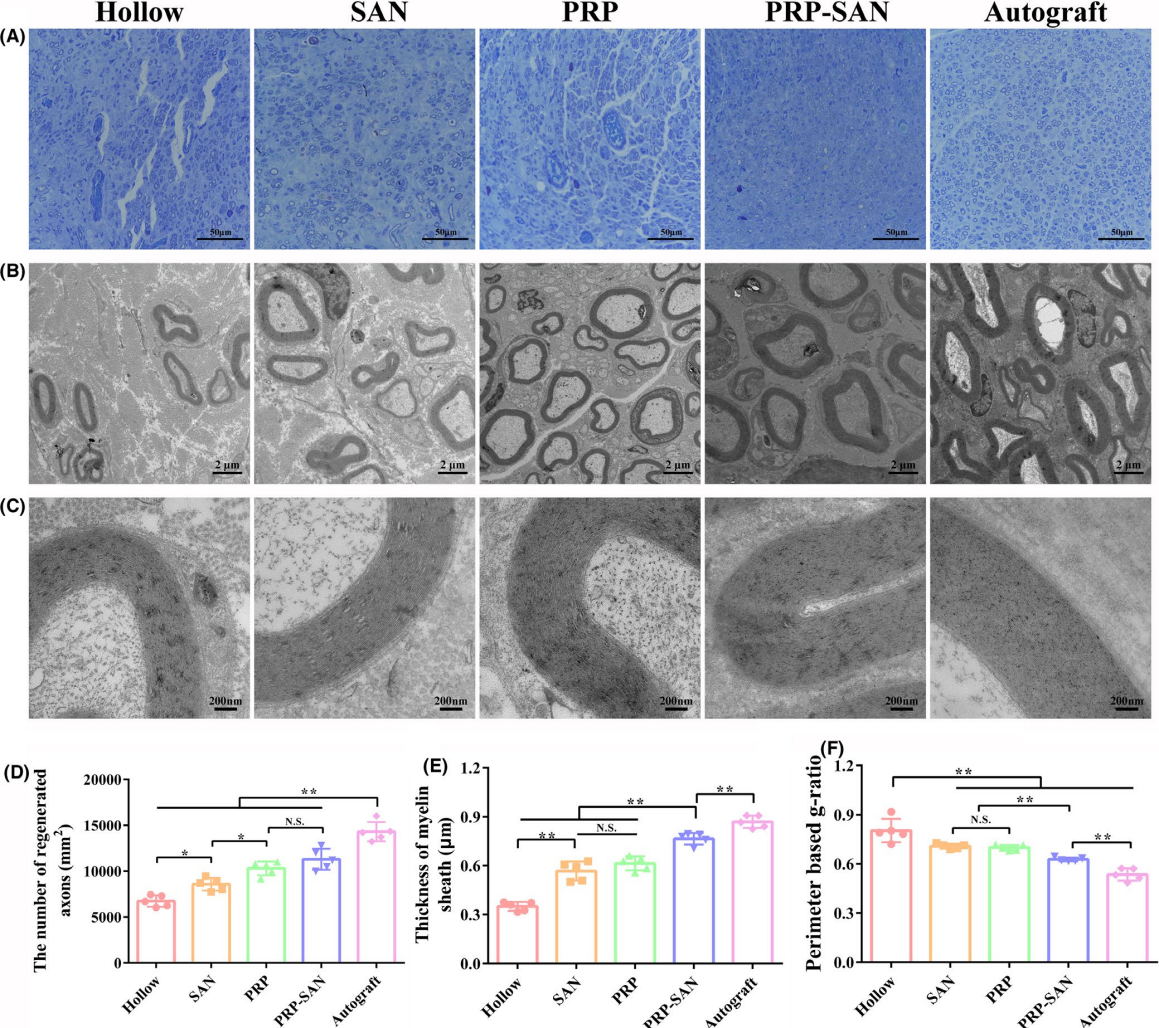
Histological evaluation of regenerated nerves at 12 weeks after surgery. (A) Representative photograph of toluidine‐blue staining for each group. Scale bar =50 μm. (B) Representative transmission electron microscopy image for each group. Scale bar =2 μm. (C) Enlargement of the image shown in (B). Scale bar =200 nm. (D) The number of regenerated axons in each group. (E) Thickness of the myelin sheath in each group. (F) Perimeter‐based g‐ratio in each group. Data are presented as the mean ±SD. ^*^ *p* <.05; ^**^ *p* <.01; N.S., no significant difference

Results of toluidine‐blue staining and electron microscopic examination of transverse sections of the rat grafts are shown in Figure 7. Figure 7A shows representative images of toluidine‐blue staining for each group and the number of myelinated nerve fibers in a unit field of vision. The results revealed that the number of regenerated myelinated nerve fibers in the PRP‐SAN group was not significantly different from that in the PRP group, but higher than those in the SAN and Hollow groups (*p *< .05), and significantly lower than that in the Autograft group (*p *< .01) (Figure 7D). Figure 7B shows representative transmission electron microscopy images of ultrathin sections of the distal grafts for each group. The myelin sheath thickness of the regenerated nerve fibers in the PRP‐SAN group was significantly higher than those in the PRP, SAN, and Hollow groups (*p *< .01) but significantly lower than that in the Autograft group (*p *< .01) (Figure 7E). In addition, the perimeter‐based g‐ratio value was calculated to further evaluate the degree of myelination of the regenerated nerve fibers. The g‐ratio of the regenerated nerve fibers was significantly lower in the PRP‐SAN group than in the PRP and SAN groups but higher than in the Autograft group. No difference was observed between the PRP and SAN groups (Figure 7F). Figure 7C shows the local enlargement of the myelin sheath of representative regenerated nerve fibers in each group.

### 3.6 Gastrocnemius muscle recovery

**FIGURE 8:**
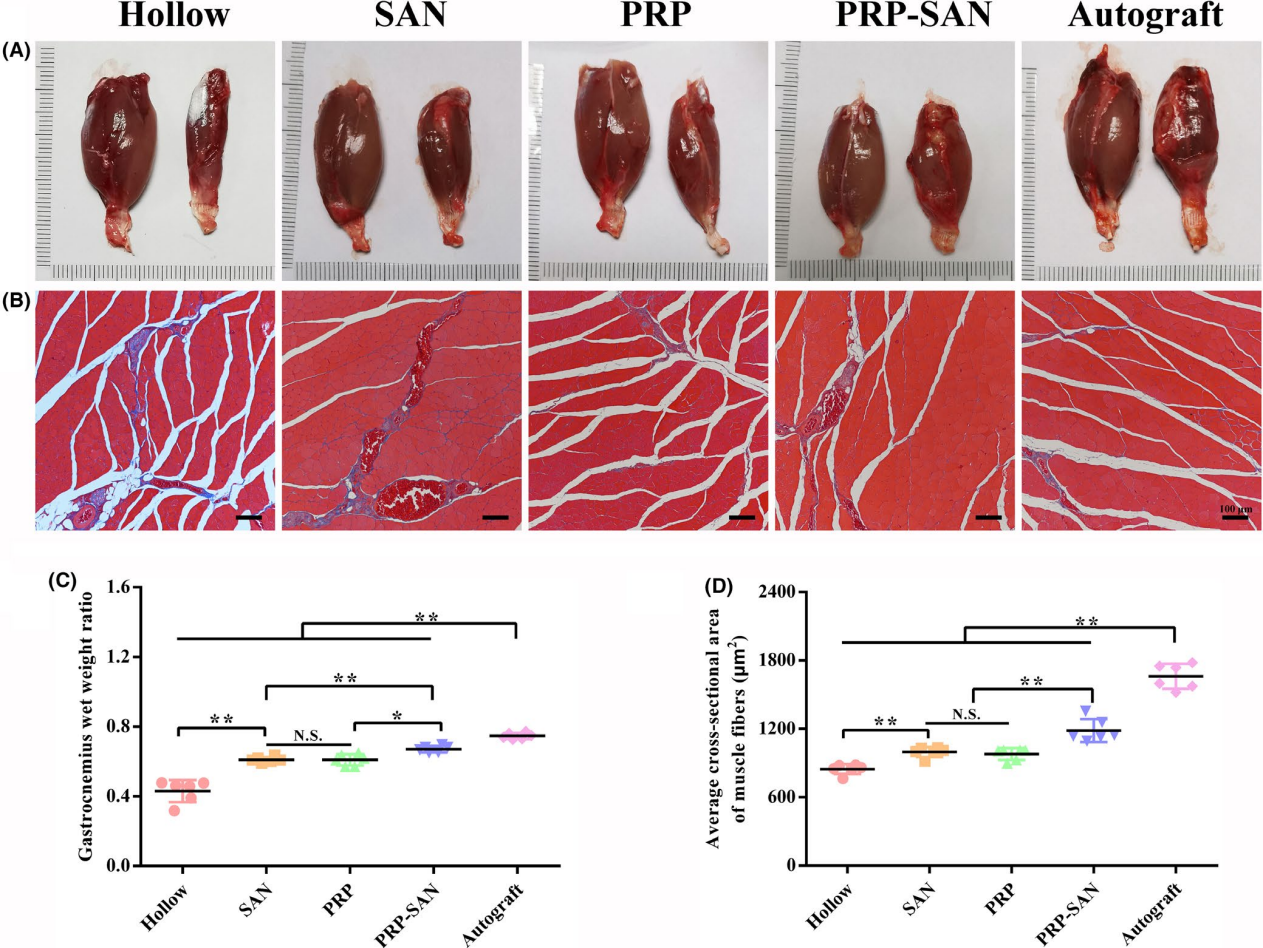
Histological evaluation of the gastrocnemius at 12 weeks after surgery. (A) Representative photograph of normal (left) and injured (right) gastrocnemius muscles. (B) Representative photograph of Masson's trichrome staining of transverse sections of an injured gastrocnemius. Scale bar =100 μm. (C) Gastrocnemius wet weight ratio (weight of injured muscle to weight of normal muscle) in each group. (D) Average cross‐sectional area of muscle fibers in each group. Data are presented as the mean ±SD. ^*^ *p* <.05; ^**^ *p* <.01; N.S., no significant difference

The gastrocnemius muscles of two rats in each group were removed and weighed at 12 weeks after nerve transplantation, and Masson's trichrome staining was performed on the affected muscles. The results are shown in Figure 8. Representative images of the affected and healthy gastrocnemius muscles in each group are shown in Figure 8A. The wet weight ratio of affected‐to‐healthy gastrocnemius muscle in the PRP‐SAN group was significantly lower than that in the Autograft group (*p *< .01), but higher than those in the PRP, SAN, and Hollow groups (*p *< .05). No difference was observed between the PRP and SAN groups (Figure 8C). In addition, Figure 8B shows representative images of Masson's trichrome staining of the middle abdominal section of the affected gastrocnemius for each group. With Masson's trichrome staining, significantly fewer blue‐stained collagen fibers were observed in the PRP‐SAN group than in the PRP, SAN, and Hollow groups. The mean muscle fiber cross‐sectional area in the PRP‐SAN group was significantly lower than that in the Autograft group (*p *< .01) but significantly higher than those in the other three groups (*p *< .01) (Figure 8D).

### 3.7 FG retrograde tracing

**FIGURE 9:**
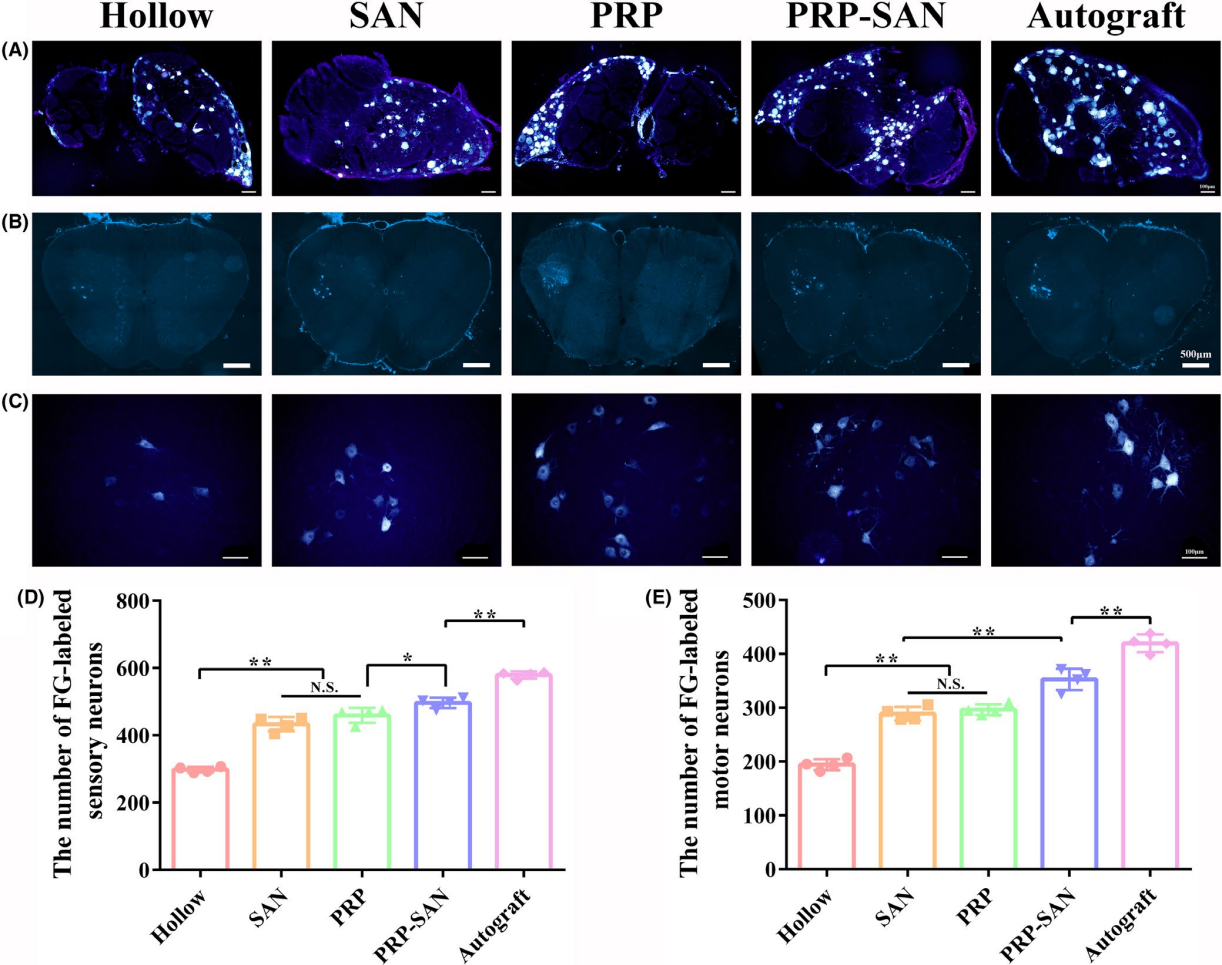
Fluor‐Gold (FG) retrograde tracing of neurons at 12 weeks after surgery. (A) Representative photograph of FG‐labeled sensory neurons in each group. Scale bar =100 μm. (B) Representative photograph of FG‐labeled motor neurons in each group. Scale bar =500 μm. (C) Enlargement of the photograph shown in (B). Scale bar =100 μm. (D) The number of FG‐labeled sensory neurons in each group. (E) The number of FG‐labeled motor neurons in each group. Data are presented as the mean ±SD. ^*^ *p* <.05; ^**^ *p* <.01; N.S., no significant

The numbers of DRG sensory neurons and anterior spinal motor neurons growing through the nerve defects in each group were evaluated at 12 weeks after nerve transplantation using the FG retrograde tracing method to analyze the effects of nerve grafts on nerve regeneration in each group. Figure 9A shows representative FG‐labeled sensory neurons for each group. The number of FG‐labeled sensory neurons in the PRP‐SAN group was higher than those in the PRP, SAN, and Hollow groups (*p *< .05), but significantly lower than that in the Autograft group (*p *< .01) (Figure 9D). Figure 9B shows representative FG‐labeled motor neurons for each group. The number of FG‐labeled motor neurons in the PRP‐SAN group was significantly higher than those in the PRP, SAN, and Hollow groups (*p *< .01) but significantly lower than that in the Autograft group (*p *< .01) (Figure 9E). No difference was observed between the PRP and SAN groups. Figure 9C shows a locally enlarged view of representative motor neurons at the anterior horn of the spinal cord for each group.

### 3.8 MEPs evaluation

**FIGURE 10:**
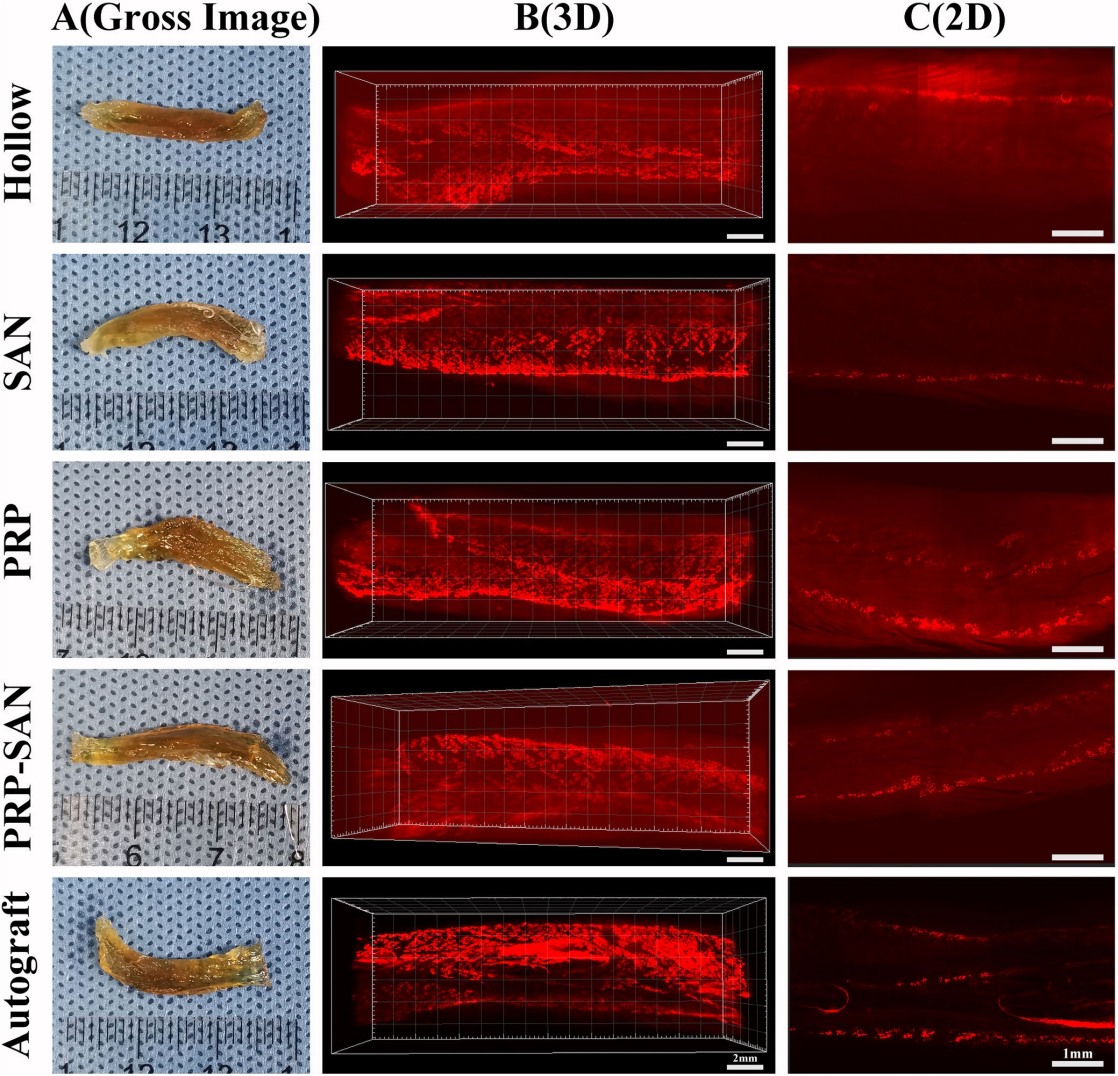
The motor endplates (MEPs) of flexor digitorum longus at 12 weeks after surgery. (A) Representative photograph of transparency of the flexor digitorum longus after 3DISCO in each group. (B) Representative three‐dimensional distributions of MEPs in flexor digitorum longus. Scale bar =2 mm. (C) The coronal plane photograph shown in (B). Scale bar =1 mm

The number of MEPs labeled with BTX 647 reagent at 12 weeks after the operation was assessed to evaluate the number of relationships established between motor nerve fibers and muscle fibers. Figure 10A shows representative transparent images of the flexor digitorum longus for each group. Figure 10B shows the three‐dimensional MEPs model of the flexor digitorum longus for each group, indicating the three‐dimensional distribution of MEPs in the muscles. Figure 10C shows the coronal plane of the three‐dimensional MEPs model of the flexor digitorum longus for each group.

## 4 DISCUSSION

Peripheral nerve injury is a common disease in clinical medicine. The current gold standard for treating peripheral nerve injuries is autologous nerve transplantation, and it has been challenging to develop a promising substitute treatment. In this study, we constructed new nerve grafts that produce better repair outcomes by combining chitin biological conduits with SANs and PRP.

Chitin biological conduits are widely used to repair peripheral nerve defects due to their good biocompatibility, mechanical properties, and degradability.^12^, ^18^ In this study, the results of staining longitudinal graft sections at 2 weeks and graft middle cross‐sections at 12 weeks after nerve transplantation revealed that regenerated nerve fibers grew successfully through the long chitin biological conduit and no inflammatory cells infiltrated. The results show that the chitin biological conduit provided reliable mechanical support for nerve growth, while the invasion of scar tissue was avoided.

In general, nerve repair outcomes are often poor due to the lack of a suitable microenvironment to promote nerve regeneration in the hollow nerve conduit. Therefore, various neurotrophic factors, such as NGF, BDNF, and GDNF, have been used to enhance the repair outcomes of peripheral nerve injury.^20^, ^21^, ^22^ Because PRP contains a variety of growth factors, many studies have investigated its effects on peripheral nerve regeneration.^28^, ^29^, ^30^ In addition, PRP becomes a gel when activated by thrombin and calcium chloride. The PRP gel has a dual effect on peripheral nerve regeneration. First, the PRP gel is gelatinous and has a structure similar to that of extracellular matrix, thus providing a scaffold for nerve growth. Second, various factors contained in PRP activate SCs, promote macrophage polarization, prevent inflammation and fibrosis, and stimulate angiogenesis to accelerate the recovery of peripheral nerve function.^31^, ^32^ In this study, PRP was prepared by two‐step centrifugation of autologous blood from rats. There is no unified and clear standard for platelet content in PRP. Platelet enrichment levels of 3–8 in PRP are considered most conducive for promoting tissue healing and repair.^33^ In this study, the platelet enrichment level of the PRP was 4.89, which is within the optimal range.

In addition, SANs are readily available during clinical surgery without causing additional sensory or motor impairments due to their extraction. SANs secrete neurotrophic factors and promote SC proliferation during Wallerian degeneration. Moreover, small nerves with a complete structure afford a hierarchically aligned structure, which provides the best bridge for the rapid growth of axons.

The nerve grafts constructed in this experiment were used to evaluate the promotion of nerve repair using a 10‐mm sciatic nerve defect rat model. The results of the histochemical evaluation at 2 weeks after nerve transplantation showed that the number of regenerated axons in the PRP‐SAN and PRP groups was greater than that in the Hollow group, while regenerated axons in the PRP‐SAN and SAN groups grew faster than those in the Hollow group. The SFI value, CMAP intensity, histological outcomes of the regenerated axons, and gastrocnemius recovery were better in the PRP‐SAN, PRP, and SAN groups than in the Hollow group at 12 weeks after the operation.

Axons can transport material both anteriorly and in a retrograde manner. The axon transport function is lost once it is damaged. If the continuity of a damaged axon can be restored, the axon transport function can also be restored. The axon transport function is an important indicator reflecting the structure, metabolism, and functional integrity of a nerve, and the restoration of axon transport function after nerve injury repair is powerful evidence for nerve regeneration.^34^, ^35^, ^36^ In this study, the distal part of the transplanted nerve was infiltrated with FG, which can be transported to the body of the neuron by way of retrograde axoplasmic transport. This allowed us to assess the number of axons recovered from axoplasmic transport in the transplanted nerve segment. The results showed that nerve axon recovery in the PRP‐SAN, PRP, and SAN groups was better than that in the Hollow group, and nerve axon recovery in the PRP‐SAN group was better than that in the PRP and SAN groups, which was consistent with expectations.

In addition, BTX 647 reagent was used in an experiment to mark MEPs, which indicate the locations where peripheral nerves exchange information with skeletal muscle. Restoration of the MEPs structure is crucial for the recovery of skeletal muscle function.^37^, ^38^ The distribution of the MEPs layer of skeletal muscle reflects the repair outcome of peripheral nerves. According to the results, the MEPs distribution in the PRP‐SAN, PRP, and SAN groups was denser than that in the Hollow group.

These results indicate that although chitin biological conduits combined with PRP alone or SANs alone can promote peripheral nerve regeneration, the combination of PRP and SANs had a greater facilitating effect than either one alone on peripheral nerve regeneration. At the same time, the SANs and PRP used in this experiment were obtained from the body in a quick, safe, and convenient manner. Therefore, the protocol described here can be a new method for peripheral nerve defect repair in the future.

## 5 CONCLUSIONS

In summary, nerve grafts comprising chitin biological conduits combined with SANs and PRP significantly promoted the repair of 10‐mm sciatic nerve defects in rats. PRP and SANs in the graft were completely self‐derived and obtained quickly and safely. These results will contribute to the development of an effective strategy for repairing peripheral nerve defects.

## DISCLOSURE

The authors declare no conflict of interest.

## AUTHOR CONTRIBUTIONS

Chang‐Feng Lu contributed to investigation, methodology, data curation, software, validation, and writing—original draft. Bo Wang contributed to project administration, resources, and formal analysis. Pei‐Xun Zhang contributed to supervision and resources. Shuai Han contributed to software and resources. Wei Pi contributed to methodology and validation. Yu‐Hui Kou contributed to supervision, writing—review and editing, and funding acquisition. Bao‐Guo Jiang contributed to supervision and funding acquisition.

## Data Availability

The data are available from the corresponding author upon reasonable request.
